# Creating a Culturally Safe Online Data Collection Instrument to Measure Vaccine Confidence Among Indigenous Youth: Indigenous Consensus Method

**DOI:** 10.2196/52884

**Published:** 2024-08-12

**Authors:** Marion Maar, Caleigh Bourdon, Joahnna Berti, Emma Bisaillon, Lisa Boesch, Alicia Boston, Justin Chapdelaine, Alison Humphrey, Sandeep Kumar, Benjamin Maar-Jackson, Robert Martell, Bruce Naokwegijig, Davinder Preet Kaur, Sarah Rice, Barbara Rickaby, Mariette Sutherland, Maurianne Reade

**Affiliations:** 1 Human Sciences Division Northern Ontario School of Medicine (NOSM) University Sudbury, ON Canada; 2 Undergraduate Medical Education Northern Ontario School of Medicine (NOSM) University Sudbury, ON Canada; 3 Debajehmujig Storytellers Debajehmujig Theatre Group Manitowaning, ON Canada; 4 Interdisciplinary Health Laurentian University Sudbury, ON Canada; 5 Public Health Sudbury & Districts Sudbury, ON Canada; 6 Matawa Health Co-operative Thunder Bay, ON Canada; 7 Cinema and Media Arts York University Toronto, ON Canada; 8 Postgraduate Medical Education Northern Ontario School of Medicine (NOSM) University Thunder Bay, ON Canada; 9 School of Nursing Laurentian University Sudbury, ON Canada; 10 School of Rural and Northern Health Laurentian University Sudbury, ON Canada; 11 Chiefs of Ontario Birch Island, ON Canada; 12 Clinical Sciences Divison Northern Ontario School of Medicine (NOSM) University Sudbury, ON Canada

**Keywords:** ATKC consensus method, community-based participatory research, COVID-19 vaccines, cultural competency, electronic survey development, Indigenous Peoples, vaccine confidence, vaccine hesitancy, youth

## Abstract

**Background:**

Participating in surveys can shape the perception of participants related to the study topic. Administering a vaccine hesitancy questionnaire can have negative impacts on participants’ vaccine confidence. This is particularly true for online and cross-cultural data collection because culturally safe health education to correct misinformation is typically not provided after the administration of an electronic survey.

**Objective:**

To create a culturally safe, online, COVID-19 vaccine confidence survey for Indigenous youth designed to collect authentic, culturally relevant data of their vaccine experiences, with a low risk of contributing to further vaccine confusion among participants.

**Methods:**

Using the Aboriginal Telehealth Knowledge Circle consensus method, a team of academics, health care providers, policy makers, and community partners reviewed COVID-19 vaccine hesitancy surveys used in public health research, analyzed potential risks, and created a framework for electronic Indigenous vaccine confidence surveys as well as survey items.

**Results:**

The framework for safer online survey items is based on 2 principles, a first do-no-harm approach and applying a strengths-based lens. Relevant survey domains identified in the process include sociodemographic information, participants’ connection to their community, preferred sources for health information, vaccination uptake among family members and peers, as well as personal attitudes toward vaccines. A total of 44 survey items were developed, including 5 open-ended items to improve the authenticity of the data and the analysis of the experiences of Indigenous youth.

**Conclusions:**

Using an Indigenous consensus method, we have developed an online COVID-19 vaccine confidence survey with culturally relevant domains and reduced the risk of amplifying misinformation and negative impacts on vaccine confidence among Indigenous participants. Our approach can be adapted to other online survey development in collaboration with Indigenous communities.

## Introduction

### Overview

The World Health Organization (WHO) declared vaccine hesitancy as one of the top 10 threats to global health in 2019 [1,2]. Although not a novel phenomenon, low vaccine uptake is said to have led to new outbreaks of previously controlled childhood diseases [3-5]. It has also been reported that vaccine refusal since the start of the COVID-19 pandemic has led to hundreds of thousands of unnecessary deaths [6-8]. Research on what leads people to refuse one of the most effective preventative measures of disease prevention is therefore of great importance to public health [6,9].

The WHO’s Strategic Advisory Group of Experts on Immunization (SAGE) Working Group on Vaccine Hesitancy defines vaccine hesitancy as the “delay in acceptance or refusal of vaccination despite availability of vaccination services” [10]. Various complex factors, such as medical mistrust and socioeconomic inequities, influence an individual’s lack of confidence in vaccinations [11-13]. A relatively new phenomenon and a growing concern for vaccine acceptance is the spreading of vaccine misinformation through social media. Interacting with social media, people participate in a transformational process as they add comments, captions, messages, and other elements, to interact with and propagate information; as they engage with information, they may internalize and repeat misinformation and further tailor misinformation messages to fit the specific audiences of their social media [14]. The audiences in turn may lack the time, resources, or level of health literacy required to independently access the veracity of the messages, which can exacerbate the impact of misinformation.

Public health studies have explored the causes of vaccine hesitancy and types of misinformation beliefs using survey research [12,15-19]. However, when interacting with vaccine perceptions on social media platforms, there is a risk of amplifying vaccine misinformation when participants are asked to rank their agreement with misinformation statements [20]. The risk is that either the participants are exposed to misinformation for the first time or to the reinforcement of preexisting beliefs [21], which can result in a backfire effect, a form of confirmation bias, wherein attempts to correct misinformation with evidence can instead strengthen belief in the misinformation [22,23]. The risk is magnified when the administration of a survey is not followed with health education, which usually is the case for electronic surveys (e-surveys). Although more research is needed on this topic, research shows that corrections need to be paired with education rather than merely labeling misinformation as false [21,24].

Thus, the development of any vaccine hesitancy survey, especially those administered online, requires special consideration of potential risks and safety. If the survey is used in an Indigenous population, further cultural-specific considerations are required including recognition of the history of medical experimentation and the existence of structural racism that has fostered mistrust toward the medical community [11-13,18,25-27]. Survey questions must be culturally relevant and safe, meaning that participants should feel respected, see their culture reflected, and not be exposed to harms such as inadvertent reinforcement of misinformation or distrust in vaccine initiatives. Researchers should also be careful to achieve equivalence of the meaning of questions in the cultural context of the participating Indigenous people [28].

### Background

Although Indigenous Peoples, especially those living in rural communities, were disproportionately affected by the COVID-19 pandemic [29,30], it is important to acknowledge the successes of Indigenous communities in leading vaccination efforts [26,31-33]. Vaccine uptake in Indigenous communities varies, and the reasons for vaccine acceptance are diverse [34]. For example, Indigenous youth and young adults in Indigenous communities in British Columbia, Canada, had significantly lower levels of vaccine acceptance than their older counterparts [35], and it is likely that the factors influencing their vaccine uptake may differ from those of older community members [36].

In contrast, in urban areas, there is inadequate quality and accessibility of health and sociodemographic statistics for Indigenous populations in Canada [37]. The absence of accurate, inclusive, and culturally appropriate identification procedures for Indigenous Peoples within health services and public health data systems, coupled with inadequate collaboration with Indigenous leadership in health data governance contribute to significant data gaps. As a result, Indigenous health service planning is frequently based on inadequate data. Culturally appropriate survey instruments could support the development of Indigenous health data.

To facilitate successful vaccine administration in Indigenous communities, Indigenous Services Canada (ISC) identified the need for a culturally safe approach to vaccine education and administration, a proactive approach to address vaccine hesitancy, as well as relying on local organizational and community advice related to local vaccine confidence and hesitancy [27]. The dissemination of culturally appropriate communications and interventions, reflective of community norms, has been demonstrated to establish trust and promote vaccine confidence among Indigenous populations [34]. Given that newly developed vaccines raise legitimate concerns, it is vital to seize these opportunities to engage with individuals and address their concerns respectfully. Engaging individuals with an open dialog and establishing trust are facilitators for vaccine promotion strategies. Furthermore, expressing empathy throughout these discussions is particularly important for overcoming barriers to vaccine confidence; especially as antivaccine rhetoric is often leveraging resistance to heavy-handed approaches to vaccine rollout [38]. Finally, respectful collaboration among health authorities, provincial or territorial governments, and Indigenous nations is paramount for fostering vaccine acceptance [39].

We hypothesized that an online vaccine survey, administered in collaboration with local community organizations, could contribute to the need for information about Indigenous Peoples’ perspectives about vaccines while keeping the high cost of conducting research in remote Indigenous communities contained and expanding uptake of the survey in urban areas. In order to support future research into vaccine hesitancy among Indigenous youth, we set out to develop a culturally based e-survey instrument tailored to Indigenous youth in Canada, with the intention of reducing the risk of amplifying vaccine misinformation.

## Methods

### An Indigenous Consensus Method for e-Survey Development

The e-survey development was part of a larger study on vaccine confidence that required the collection of baseline data on vaccine hesitancy factors among Indigenous youth. The survey instrument was developed in this phase of the study, using an Indigenous methodology, and the Aboriginal Telehealth Knowledge Circle (ATKC) consensus method. The ATKC consensus method was designed to reach an agreement on eHealth initiatives in Indigenous communities [40]. In contrast to Western consensus methods such as the Delphi method [41], the ATKC approach “shifts the focus from expert panels to communities of practice and policy-makers; individuals with a lived experience and deep understanding of… [Indigenous] communities, e-health applications, policies, and politics” [40]. Thus, voices from community members, service providers, and youth featured much more prominently in this process when compared with other more strictly academic consensus approaches such as the Delphi method.

### Consensus Process

In line with the ATKC approach, our consensus research team was composed of community partners (n=4), providers (n=3), researchers (n=5), and health care and graduate students (n=5). Our partners included the Indigenous public health sector, Indigenous arts-based organizations, health care providers, planners, educators, consultants who work with local Indigenous communities, Indigenous youth, students in health-related programs, and academic researchers. A total of 17 individuals participated in the consensus process.

The academic members conducted a literature review and collected published COVID-19 vaccine hesitancy questionnaires [15-17,19,20,34,42-50], which were shared with the rest of the team. In accordance with the ATKC approach, our consensus group did not use a majority rule, instead “consensus was considered to be achieved when no new ideas or issues were tabled during a discussion and participants voiced agreement” [40], with the appropriateness of each survey item. A total of 5 rounds of consensus meetings were held to create a culturally safe questionnaire based on the perspective of the consensus team. First, the consensus team critically reviewed the selected academic and gray literature and published COVID-19 survey instruments with the objective of identifying characteristics and values associated with a culturally safe Indigenous vaccine hesitancy survey. The group also identified factors that might influence vaccine hesitancy and confidence in Indigenous communities and developed survey domains for a culturally safe Indigenous youth vaccine confidence survey. Finally, several team members met to draft survey items that were then reviewed and edited by the rest of the consensus team using this Indigenous consensus process.

### Ethical Considerations

The development of the survey instrument reflects the collaborative work of the coauthors and there were no research participants as defined by the Tri-Council Policy Statement, thus the Laurentian University Ethics Board exempted the requirement for human subject ethics review for the survey development work described in this paper.

## Results

### Consensus Characteristics and Values for a Culturally Safe e-Survey

Our group reached the consensus that the guiding values of the development of survey items should include avoiding the potential to cause harm to participants in order to not increase anxiety or misinformation, and it should apply a strengths-based lens.

### First, Do-no-Harm

The group reached the consensus that the instruments reviewed [15-17,19,20,34,42-50] did not demonstrate adequate levels of cultural safety for use with Indigenous participants. They concluded that some of the survey items might even give credence to vaccine misinformation by asking the participants to rank their agreement with beliefs about various hypothetical vaccine side effects. We understand there is interest in perceptions about vaccine-related misinformation and cognitive biases [38]; however, we also believe that when delivered as an e-survey and without concurrent health education, vaccine misinformation could be amplified. This in turn could lead to increased risk of harm to participants by increasing their vaccine concerns and thus lowering vaccination rates and increasing preventable disease.

In seeking to understand barriers and enablers to vaccine acceptance, it is important to design surveys in a way that does not introduce more vaccine hesitancy as an unintended consequence. For example, open-ended nonleading questions such as “Do you have any concerns about the COVID-19 vaccine?” were preferred by the consensus group over asking about concerns with specific side effects of the vaccine. Our team also sought to frame questions in such a way as to avoid increasing polarizing beliefs or conspiratorial ideas.

### Strengths-Based Lens

O’Keefe et al [51] argue that an “Indigenous strengths-based approach to health and wellness research” has yet to be developed. However, our group articulates a strengths-based survey design to include measures of protective factors such as connection to community, culture, and land. The consensus team identified that a focus on cultural safety, community strengths, and community self-determination was lacking in existing surveys, even though these are, according to the literature, important mediators of vaccine uptake in Indigenous communities [34]. Ignoring these mediators could also be harmful because the lack of culturally safe vaccine services can reduce the uptake of vaccines. In fact, a recent scoping review supports the importance of trusted community-based information sources as mediators of vaccine confidence among Indigenous Peoples [34]. Perceptions regarding the comfort with and cultural safety of health services were therefore thought to be important issues to be explored in the survey.

The consensus team also prioritized additional strengths-based perspectives in the design of survey items. A strengths-based survey item orientation was preferred over the more common deficit-focused scoring of, for example, health knowledge, attitudes, and beliefs. As part of the strengths-based approach, the group therefore also shifted the orientation from vaccine hesitancy to vaccine confidence. As described by Corbie-Smith, the use of the term “vaccine hesitancy” may shift the blame onto individuals and ignore important factors, such as structural racism, which are contributing to low vaccine uptake [25]. In contrast, vaccine confidence is defined as the level of trust people have in the safety and effectiveness of vaccines, the systems that facilitate the delivery of vaccines, and the motives of vaccine policy makers [52]. This shift helped the creation of strengths-based survey items by, for example, identifying sources and conditions that lead to positive attitudes toward vaccination in order to improve vaccine uptake instead of focusing on barriers.

### Consensus Domains for an Indigenous Vaccine Confidence e-Survey

After the group had reached a consensus on guiding values, the members identified survey domains of required data to ascertain vaccine confidence. The domains are composed of sociodemographic information, participants’ connection to their community, their preferred sources for health information, vaccination uptake among family members and peers, as well as their personal attitudes toward vaccines. Once the survey values and domains were established, we drafted the survey items and reached a consensus over the course of 3 meetings with concurrent email discussions with the consensus team.

### Consensus Survey Items for an Indigenous Vaccine Confidence e-Survey

Based on the values and relevant domains, a culturally safe vaccine confidence survey was developed. The group reached the consensus that the resulting survey instrument lends itself well to online delivery. A discussion of each domain and the survey items are provided next.

### Sociodemographic Profile

Questions 1-3 of the survey provide sociodemographic data that includes age, gender, and place of residence.

### Connection to Community

This domain was created because studies have shown that strong Indigenous community connections and community-driven interventions are effective at improving vaccine confidence [17,25,38,53]. Successful communication strategies prioritize the protection of older adults, community, and culture, leveraging Indigenous knowledge and traditions to improve vaccine uptake [31-33,54,55]. This includes integrating messaging from trusted figures such as community leaders, respected older adults, and Indigenous health care professionals. Indigenous Peoples have incorporated cultural insights and information from tribal health authorities when responding to the threat of COVID-19, which underscores the significance of Indigenous perspectives in comprehending health communication and risk assessment [55].

Questions 5-10 explore community connection using the Native Wellness Assessment Self-Report Form (NWA-S Tool) [56], a validated psychometric instrument that measures a sense of belonging, contribution to the community, strengths of Indigenous communities, and individuals’ relationships to Indigenous culture and the land [57]. Questions 12 and 13 were included to specifically assess community involvement through volunteering, distinct from other ways of contributing to one’s community (question 6). Volunteering influences a sense of community belonging, support and well-being, and indeed is predictive of well-being [58]. The Native Women’s Association of Canada explains the inherent nature of volunteerism within First Nations communities, and that informal volunteerism is much more common, usually seen as “helping out” [59].

### News and Media Sources–Health Information

Communication approaches and social media are key influencers in shaping vaccine confidence [1], while the high prevalence of misinformation poses a significant challenge to vaccine confidence [2,60-67]. In exploring the factors influencing vaccine confidence, it is important to distinguish between scientific and narrative modes of thinking. Scientific evaluations rely on empirical evidence, whereas narratives are judged subjectively by people based on their perceived authentic portrayal of human experiences [68]. Individuals engage with narratives based on their trust in the source of information, and once trust is established, it furthermore amplifies the likelihood of sharing narratives [14]. Hence, there is a necessity for trusted messengers and credible sources of information in disseminating public health information to ensure public health goals are met. Questions 14-22 from this section may provide valuable insights into understanding the mechanisms through which Indigenous youth approach scientific and narrative modes of thinking related to vaccines. The items also shed light on how misinformation spreads and influences vaccine acceptance among Indigenous youth, as well as identify the appropriate media channels to strengthen vaccine confidence.

### Vaccination Uptake Among Individuals, Family, or Friends

High vaccine hesitancy is generally associated with low vaccine uptake and this has been true during the COVID-19 pandemic as well as historically [38,65]. High uptake of vaccines was essential for the eradication of many illnesses such as polio and smallpox [65]. However, in recent years, due to decreasing vaccination rates, some of these vaccine-preventable diseases, such as measles, have re-emerged [3,5,65,69]. Some studies have shown associations between uptake of other vaccinations (such as the seasonal influenza vaccine) and COVID-19 vaccines [65,70,71]. In addition, other studies have shown that friends and family are an important influence on decisions to be vaccinated [53,62,72,73]. Thus, survey questions 23-33 include a general exploration of the uptake of vaccination among close circles and social pressures.

### Attitudes and Beliefs Concerning Vaccines

Vaccine confidence among Indigenous Peoples is a complex issue shaped by historical and contemporary contexts [13]. Attitudes and beliefs toward vaccinations vary significantly based on factors such as geographic region, age, gender, ethnicity, educational attainment, social relationships, and socioeconomic status [74]. Messages must be personalized to resonate with individual life experiences and be easily understandable for informed decision-making, especially within marginalized communities that may not readily engage with campaigns targeting the general public [75]. Recent studies have therefore advocated for tailored messaging strategies that address community-specific needs [13,31,32,55,75-77]. Cocreating culturally relevant health communication messages is therefore vital for promoting preventive behaviors and enhancing health and well-being among Indigenous populations [32,55]. This examination of personal attitudes is elicited within items 34 to 44, which are designed to shed light on the unique challenges and opportunities in promoting COVID-19 vaccination within Indigenous communities.

### The Online Survey Domains and Items

The finalized list of survey domains and survey items is provided in Table 1.

**Table 1:** Final list of survey domains and items included in the Indigenous youth vaccine confidence survey.

Domain and number		Survey item	Responses
**Section A: sociodemographic profile**			
	1	Please select your age	Dropdown list of ages 16-29 years
	2	What is your gender?	Male Female Non-binary Other Prefer not to specify
	3	Where do you live? (place of residence or community)	Open-ended
**Section B: connection to community**			
	4	I feel a sense of belonging in my community.	Strongly agree Agree Neither agree nor disagree Disagree Strongly disagree Don’t know
	5	I feel confident getting support from my community.	Strongly agree Agree Neither agree nor disagree Disagree Strongly disagree Don’t know
	6	I recognize that I can contribute to my community.	Strongly agree Agree Neither agree nor disagree Disagree Strongly disagree Don’t know
	7	I see the strengths Indigenous Peoples have as a community.	Strongly agree Agree Neither agree nor disagree Disagree Strongly disagree Don’t know
	8	I feel a connection between my community history and my own story or life.	Strongly agree Agree Neither agree nor disagree Disagree Strongly disagree Don’t know
	9	My relationship to the land I come from is important.	Strongly agree Agree Neither agree nor disagree Disagree Strongly disagree Don’t know
	10	My language and a connection to the land help me to know who I am.	Strongly agree Agree Neither agree nor disagree Disagree Strongly disagree Don’t know
	11	I generally trust the advice of my elders.	Strongly agree Agree Neither agree nor disagree Disagree Strongly disagree Don’t know
	12	I am regularly involved in formal volunteer activities, such as being a member of a group or a school committee.	Strongly agree Agree Neither agree nor disagree Disagree Strongly disagree Don’t know
	13	I volunteer for my community in other ways, such as helping out with elders or activities for children.	Strongly agree Agree Neither agree nor disagree Disagree Strongly disagree Don’t know
**Section C: news and media sources - health information**			
	14	Which news or social media platforms do you get your news from? (Check all that apply)	YouTube Instagram Twitter Local Facebook groups Facebook groups outside of my community TikTok News sites (if so, please indicate which ones in the comment box below) School (teachers, principals) Family members Other (please specify in the comment box below)
	15	Which news or social media platforms do you use the most to inform yourself? (Please identify your top 3)	YouTube Instagram Twitter Local Facebook groups Facebook groups outside of my community TikTok News sites (if so, please indicate which ones in the comment box below) School (teachers, principals) Family members Other (please specify in the comment box below)
	16	How often do you and your family members discuss the news?	Often Usually Sometimes Rarely Never
	17	How often do you and your friends discuss the news?	Often Usually Sometimes Rarely Never
	18	How often do you and your family members discuss the COVID-19 pandemic?	Often Usually Sometimes Rarely Never
	19	How often do you and your friends discuss the COVID-19 pandemic?	Often Usually Sometimes Rarely Never
	20	How often do you use multiple sources to learn about the news?	Often Usually Sometimes Rarely Never
	21	How do you verify a claim you hear on the news?	Open-ended
	22	What do you do when you’re unsure about what you hear on the news?	Open-ended
**Section D: vaccination uptake among individuals, family, or friends**			
	23	In general, would you say your health is…	Excellent Very good Good Fair Poor Don’t know
	24	To the best of your knowledge, have you received your childhood vaccines?	Yes, all/ most No, few/ none Don’t know Prefer not to answer
	25	Have you received the annual flu shots?	Yes, every year Yes, some years No, never Don’t know Prefer not to answer
	26	To the best of your knowledge, have your family members received their annual flu shots?	Yes, every year Yes, some years No, never Don’t know Prefer not to answer
	27	To the best of your knowledge, have your friends received their annual flu shots?	Yes, every year Yes, some years No, never Don’t know Prefer not to answer
	28	To the best of your knowledge, have your family members received their childhood vaccinations?	Yes, every year Yes, some years No, never Don’t know Prefer not to answer
	29	To the best of your knowledge, have your friends received their childhood vaccinations?	Yes, every year Yes, some years No, never Don’t know Prefer not to answer
	30	How many of your family members have been vaccinated against COVID-19?	All or most of my immediate family members Some or a few of my immediate family members None of my immediate family members Don’t know Prefer not to answer
	31	How many of your friends have been vaccinated against COVID-19?	All or most of my friends Some or a few of my friends None of my friends Don’t know Prefer not to answer
	32	Have you received the COVID-19 vaccine?	Yes, 1 dose Yes, 2 doses Yes, 3 or more doses No Prefer not to answer
	33	Were you required to get the COVID-19 vaccine to return to school or work?	Yes No Don’t know Prefer not to answer
**Section E: attitudes and beliefs concerning vaccines**			
	34	What is your general attitude or belief about the vaccine? (Check all statements you agree with)	I am willing to get or have already taken the vaccine I am willing to take more vaccine booster shots in the future I am willing to recommend the vaccine to friends and family I am reluctant to take the vaccine I am reluctant to take more vaccine booster shots in the future I am reluctant to recommend the vaccine to friends and family I would actively discourage my friends and family from getting the vaccine I would like trusted Elders to help me make a decision about the vaccine I am okay with getting a needle if it is needed to keep me healthy I do not like needles and tend to avoid getting a needle at all costs I think getting the vaccine is painful and avoid it for that reason
	35	What is your general attitude or belief about protection relating to the vaccine? (Check all statements you agree with)	I am taking the vaccine to protect myself I am taking it to protect my family I am taking it to protect my community I am willing to take the vaccine to protect our elders
	36	Did or do you feel social pressure to get the COVID-19 vaccine?	Yes Somewhat No Don’t know Prefer not to answer
	37	Did or do you feel social pressure to not get the COVID-19 vaccine?	Yes Somewhat No Don’t know Prefer not to answer
	38	Do you believe that vaccination is important to stop the spread of COVID-19?	Yes Somewhat No Don’t know Prefer not to answer
	39	What gives you confidence or has made you trust the COVID-19 vaccine?	Open-ended
	40	Do you have any concerns about the COVID-19 vaccine?	Open-ended
	41	Where do you get your health care?	First Nation community clinic Clinic outside of community Walk-in clinic Hospital Other (please specify in the comment box below)
	42	How much do you trust your health care providers?	I trust them completely I trust them a lot I trust them somewhat I don’t trust them very much I don’t trust them at all
	43	I am interested in learning more about vaccines and how they work.	Yes No Unsure
	44	I am interested in learning more about COVID-19.	Yes No Unsure

## Discussion

### Principal Findings

We set out to create an electronic, culturally grounded, and culturally safe COVID-19 vaccine confidence survey instrument with a low risk of amplifying vaccine misinformation tailored to the realities of Indigenous youth experiences in Canada. An Indigenous research approach, the ATKC consensus process, was successfully used by our research team composed of a community of practice, including community members, service providers, and academics. Our guiding principles for survey development were a focus on doing no harm and applying a strengths-based approach.

The commitment to doing no harm and strengths-based approach had the unintended consequence that some of the questions had to be designed for open-ended data collection. An Australian study exploring the authenticity of Indigenous People’s data collected through surveys also found that restricting survey answers to a limited range of responses was seen as problematic by Indigenous study participants [28]. Indigenous people discussed the challenge of ensuring equivalency of the meaning of survey questions in their cultural context and summarized their sentiment for authentic data collection as “We need to be heard and have our reality understood” [28]. The inclusion of open-ended items is one way to have realities heard and better understood in survey-based research. However, it is important to note that data analysis is much more resource-intensive for open-ended questions when compared with scaled items. If this approach is used with a large number of participants, the use of artificial intelligence in the analysis could be explored. While our survey instrument is specifically tailored to understand perspectives related to COVID-19 vaccines, the ATKC consensus process, as well as the first do-no-harm, cultural, and strengths-based design approach can be used to inform the development of other e-survey instruments, particularly those aimed for use by Indigenous participants.

The domains were chosen to understand the context of participants’ perspectives related to COVID-19 vaccines; their sources of health information, the influence of culture, place, friends, and family members, and to better understand attitudes and behaviors related to vaccine hesitance and acceptance. Our domains also included items from the NWA tool, which have been validated to measure Indigenous youth perspectives on well-being [56]. We specifically chose those NWA items that measure a sense of community connection, because community connection has been shown to be strongly aligned with vaccine confidence in Indigenous communities [17,25,38,53]. In a recent study, Indigenous women, particularly women authority figures in communities, were noted as significant influencers in health decisions [54]. Through the use of media, Indigenous women assume a primary role in shaping narratives about the impact of the pandemic on their communities and lands [78]. Given this influential role, the particular role of Indigenous women in influencing the participant’s vaccine confidence could also be explored in vaccine confidence surveys.

Research across diverse age groups and socioeconomic neighborhoods shows that “the willingness to engage in community-related prosocial normative behavior that it positively predicts is itself an important positive predictor of COVID-19 vaccine willingness” [79]. The perceived sense of duty to the community that is connected to vaccine willingness could be linked with the ability to contribute to the community (question 6) but can also be further understood by exploring experience with informal and formal volunteerism (questions 12 and 13). Locally based volunteering is tightly connected with community identity, where volunteers identify a desire to contribute to the community through a sense of duty, pride, and obligation [58]. Discussions of prosocial behaviors in adolescents often focus on formal volunteer contributions [80,81], and yet understanding community work both “within and without an organizational context” is important [82]. Indeed, the Native Women’s Association of Canada has indicated the link between volunteerism and the concept of “helping out” to be a best practice for engaging volunteers [59].

### Limitations

The Indigenous Peoples residing within Canada include the Métis, Inuit, and First Nations cultural groups. The instrument was developed in collaboration with the First Nations Anishinaabe people, comprised of 3 related nations (Ojibwe, Odawa, and Potawatomi). Given the diversity of worldviews and adherence to traditional culture among Indigenous Peoples even within Canada, the results may be limited in their generalizability to Indigenous Peoples in other regions. However, First Nations people have important historic and cultural commonalities with other Indigenous People and thus many of the survey items will likely resonate with other Indigenous groups.

### Conclusions

Using an Indigenous consensus method, our team developed an e-survey instrument specifically tailored to measure vaccine confidence among Indigenous youth with culturally safe and strengths-based domains that can be adapted for survey research with Indigenous Peoples more generally. The team succeeded in reducing potential threats of increasing vaccine hesitancy and misinformation connected to participation and thus the survey is a more responsible and arguably safer approach to electronic delivery, especially in rural and remote locations where it may not be possible to provide vaccine education at the time of survey completion.

## Abbreviations

ATKC: Aboriginal Telehealth Knowledge Circle
ISC: Indigenous Services Canada
NWA-S Tool: Native Wellness Assessment Self-Report Form
SAGE: Strategic Advisory Group of Experts on Immunization
WHO: World Health Organization

## Data Availability

Data sharing is not applicable to this article as no data sets were generated or analyzed during this study.
